# Factors that influence the severity of post-stroke depression

**Published:** 2017

**Authors:** S Ilut, A Stan, A Blesneag, V Vacaras, S Vesa, L Fodoreanu

**Affiliations:** * Department of Neurology, “Iuliu Hațieganu” University of Medicine and Pharmacy, Cluj-Napoca, Romania; ** Department of Pharmacology, “Iuliu Hațieganu” University of Medicine and Pharmacy, Cluj-Napoca, Romania; *** “Iuliu Hațieganu” University of Medicine and Pharmacy, Cluj-Napoca, Romania

**Keywords:** NIH stroke scale, stroke, post-stroke depression, frontal lesion

## Abstract

Aim: The aim of this paper was to investigate whether the extent of neurological impairment, the location of ischemic lesions due to stroke are associated with the severity of post-stroke depression.

Materials and methods: The study included 82 patients, who were diagnosed with acute ischemic stroke and post-stroke depression and were admitted to the Neurology Clinic of Cluj-Napoca County Emergency Hospital between 2009 and 2011. A head MRI was performed with a 1.5 Tesla. Psychometric assessment was performed by using several scales, including the Beck Depression Inventory and the Mini-Mental State Examination. The National Institutes of Health Stroke Scale (NIHSS) and the Barthel Index of Activities of Daily Living were used to produce a complete neurological assessment.

Results: Patients with severe depression had a lower score on the Quality of Life Scale (QOLS) and higher scores for the Barthel index, NIHSS and MMSE. A stroke located in the basal nuclei increased the probability of severe depression. The patients with fewer lesions (1-2) had a greater chance of developing mild or moderate depression compared to the patients with 3-4 lesions. A frontal localization of the stroke was almost twice as common in patients with severe depression. If the stroke affected the left hemisphere, there was a higher probability of severe depression. In multivariate analysis, a basal nuclei lesion, a left hemisphere stroke location, and an NIHSS score >11 were all independently associated with severe depression.

Conclusion: The location of the stroke and the NIHSS score could be related to the severity of post-stroke depression.

**Abbreviations**: NIHSS = The National Institutes of Health Stroke Scale; QQL = Quality of life Scale; BDI = Beck Depression Inventory; MMSE = Mini-Mental State Examination; PSD = Post-stroke depression; MRI = Magnetic resonance imaging

## Introduction

Post-stroke depression (PSD) is a severe neuropsychiatric consequence of stroke, without clear pathogenesis or therapeutic approaches.

The great focus on this disease in contemporary research is fully justified because of the negative impact it exerts on cognitive function [**1**], physical recovery [**2**,**3**] and post-stroke survival [**4**] if the depression is not identified and treated in a timely manner.

More than 30 years have passed since the first accounts of post-stroke depression [**5**], during which time a huge amount of polymorphic observational data were collected. However, the connections between stroke aspects, such as localization and lesion volume, and the risk of developing post-stroke depression or its severity remain enigmatic.

In one study (The Perth Community Stroke Study) [**6**] conducted on 294 patients in the first 4 months after stroke, the prevalence of post-stroke depression was 23%, with 15% experiencing severe depression and 8% experiencing mild depression. There was no significant gender difference at four months; however, after 1 year, 56% of the men and 30% of the women remained depressed. In men, a severe depressive episode (40%) occurred more frequently than a mild depressive episode (16%). The opposite was observed in women, with a mild depressive episode (18%) occurring more frequently than a severe depressive episode (12%).

Stanfil et al. and Kapoor A et al. [**7**,**8**] have shown that the third patient with stroke has the risk of developing depressions of different degrees.

At the end of the two-year follow-up period, approximately 20% of the patients had died, and the disease was still present in half of those who survived [**9**]. A timely diagnosis is important, as depression can have a significant negative impact on functional recovery after stroke.

Numerous risk factors for the development of post-stroke depression have been identified, including female gender, a personal history of depression [**10**] or mental illness, the presence of other important comorbidities (for example diabetes, low vitamin D) [**11**,**12**], certain localizations of the stroke, low socioeconomic status, the existence of a spouse [**13**], inability to return to work [**14**], Latino ethnicity [**15**], increased stress response [**16**], smoking [**17**], P2X4R deletion [**18**] and the severity of neurological impairment.

The aim of this paper was to elucidate the associations between the severity of depression and the location of the stroke, the volume of ischemic lesions, and the extent of neurological impairment.

## Materials and Methods

This was an observational, analytical, cross- sectional, prospective, cohort study.

The study included 82 consecutive patients, 20 women (24.4%) and 62 men (75.6%), diagnosed with acute ischemic stroke and post-stroke depression. The median age of these patients was 66.5 (58.7-74) years.

Patients in this study were selected from those admitted to the Neurology Clinic of Cluj-Napoca County Emergency Hospital between 2009 and 2011.

The study protocol was approved by the Ethics Committee of “Iuliu Hatieganu” University of Medicine and Pharmacy. Each of the patients included in the study signed the informed consent form.

The inclusion criteria were the following: the patient was over 18 years old, a diagnosis of ischemic stroke according to the Guidelines for Diagnostic and Treatment in Neurology by using clinical and imaging criteria, and a diagnosis of depression established in the first 2 weeks after ischemic stroke.

The exclusion criteria were the following: the patient refused to sign the informed consent form, a patient history of depression or mental health treatment, hemorrhagic stroke, or the presence of aphasia.

The patient’s demographics (age, gender, origin) were recorded.

A head MRI was performed with a 1.5 Tesla magnetic resonance imaging scanner and the location and cumulative volume of acute lesions were described. The presence and extent of cerebral atrophy and any patient history of other ischemic lesions were also determined.

Psychometric assessment of mood disorders comprised of the Beck Depression Inventory (BDI), which is among the first scales specifically meant to measure the severity of depression and is commonly used as a scale in clinical research. A BDI score over 30 was associated with a severe depression. The Mini-Mental State Examination (MMSE) was used for a cognitive assessment. This scale was used to identify any cognitive dysfunction and to determine whether there is a correlation between the cognitive dysfunction or cerebral dysfunction and the severity of depression. The QOLS evaluated the patients’ perception of their quality of life on a scale of 0 to 100. The National Institutes of Health Stroke Scale (NIHSS) was used together with the Barthel Index of Activities of Daily Living to produce a complete neurological assessment.

The statistical analysis was performed by using MedCalc Statistical Software version 17.6 (MedCalc Software bvba, Ostend, Belgium; http://www.medcalc.org; 2017). Quantitative data were tested for normality of distribution by using the Kolmogorov-Smirnov test, and then the data were described by median and 25-75 percentiles. Qualitative variables were described by using the frequency and percentage. A chi-square test was used for qualitative comparisons, and a Mann–Whitney test was used for quantitative comparisons. The AUROC was used to provide a cut-off value for the NIHSS score that could predict the severity of depression. The cut-off value was chosen and was the sum of sensibility and sensitivity that was the highest. A multivariate binary logistic regression was used for assessing the independent association for some variables with severity of depression (enter method). The severity of depression was considered the dependent variable. The independent variables that were introduced in the regression were selected from those that achieved statistical significance in the univariate analysis. A p-value lower than 0.05 was considered statistically significant.

## Results

The relationship between the severity of depression and several quantitative measures is described in **Table 1**. Even though we did not find a statistically significant association between age and the severity of depression, a trend was observed: people with severe depression tended to be older than those with mild or moderate depression. The same trend was found for the lesion volume: severe depression was associated with a larger volume compared to mild depression. Patients with severe depression had a lower score on the QOLS and higher scores for the Barthel index, NIHSS and MMSE.

**Table 1:** Characteristics of quantitative variables in patients with mild/ moderate or severe depression

Variable	Mild or moderate depression	Severe depression	P
Age	64 (57; 74)	70 (61.2; 77.2)	0.1
Lesion volume	9.1 (2.5; 26.3)	16.3 (4.7; 35.5)	0.2
QOLS	70 (58.7; 80)	30 (26.2; 38.7)	<0.001
Barthel index	85 (75; 90)	67.5 (47.5; 80)	<0.001
NIHSS	8 (6.7; 11)	15 (11.2; 18.7)	<0.001
MMSE	27 (24; 29)	25.4 (19; 27.7)	0.08

The relationships between the severity of depression and several qualitative measures are described in **Table 2**. A stroke located in the basal nuclei increased the probability of severe depression. The patients with 1-2 lesions had a greater chance of developing mild or moderate depression compared to patients with 3-4 lesions. A frontal localization of the stroke was almost twice as frequent in patients with severe depression.

**Table 2:** Characteristics of qualitative variables in patients with mild/ moderate or severe depression

Variable	Mild or moderate depression	Severe depression	P
Gender	F	15 (24.2%)	5 (25%)	1
	M	47 (75.8%)	15 (75%)	
Environment	R	24 (38.7%)	10 (50%)	0.5
	U	38 (61.3%)	10 (50%)	
Frontal lesion		17 (27.4%)	10 (50%)	0.1
Prefrontal lesion		20 (32.3%)	9 (45%)	0.4
Basal nuclei lesion		29 (46.8%)	15 (75%)	0.03
Temporal lesion		25 (40.3%)	7 (35%)	0.8
Occipital lesion		13 (21%)	3 (15%)	0.7
Parietal lesion		8 (12.9%)	2 (10%)	1
Number of lesions	1-2	51 (82.3%)	12 (60%)	0.06
	3-4	11 (17.7%)	8 (20%)	
Hemisphere	R	30 (48.4%)	5 (25%)	0.1
	L	32 (51.6%)	15 (75%)	
Lacunar lesions		31 (50%)	12 (60%)	0.6
Cerebral atrophy		28 (45.2%)	10 (50%)	0.9

**Fig. 1:**
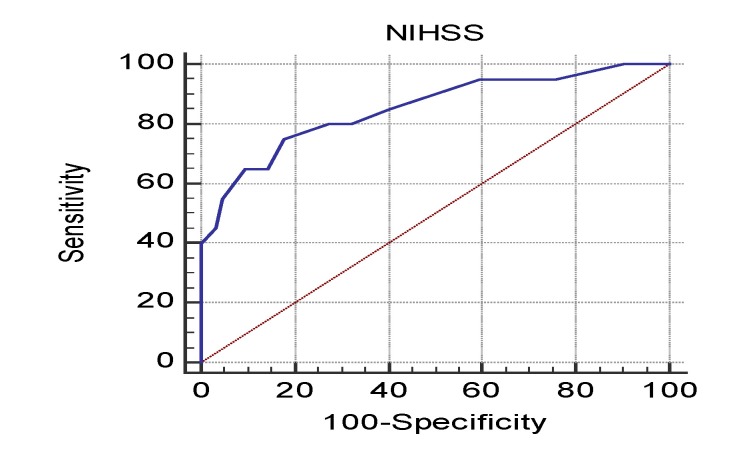
ROC curve for NIHSS score and severe depression

An AUROC was used to determine the cut-off value for the NIHSS that could predict a higher probability of severe depression. We calculated that patients with an NIHSS score over 11 were more likely to experience severe depression (AUC 0.851; Se 75%; Sp 82.2%; p<0.001; **Fig. 1**).

Several predictive models using a logistic regression were built to find the independent association of certain variables with the severity of the depression. Considering the small number of patients in our study, we included only five variables (**Table 3**). The most stable model was the one that included the presence of a frontal lesion, a lesion in the basal nuclei, a left hemisphere location, a lesion volume and an NIHSS score over 11. A lesion in the basal nuclei, a left hemisphere location and an NIHSS>11 were independently associated with the chance of severe depression.

**Table 3:** Multivariate binary logistic regression for severe depression

Variable	B	P	OR	95% C.I. for OR Min	Max
Frontal lesion	1.403	0.06	4.069	0.922	17.961
Basal nuclei lesion	1.588	0.03	4.894	1.143	20.959
Left hemisphere	1.401	0.05	4.060	1.003	16.439
Lesion volume	-0.002	0.8	0.998	0.985	1.012
NIHSS>11	2.242	0.001	9.412	2.533	34.968

## Discussions

Our study showed that several parameters could be used to predict the severity of post-stroke depression. Some specific stroke locations were strongly associated with severe depression.

After applying the inclusion and exclusion criteria, 82 patients were selected for the study. Patients were aged between 34 and 85 years, with the average age of patients with mild or moderate depression being 64 years and the average age of patients with severe depression being 70 years. Whyte et al. reported that among 65-year-olds, the risk of developing depression in less than 2 years was 6 times higher in those who had a stroke than in their non-stroke counterparts [**19**]. Depression was diagnosed by using the Beck Depression Inventory, this method being used by other studies [**20**,**21**].

In our study, the majority of the patients were male in both the mild/ moderate depression group (75.8%) and the severe depression group (75%). Mbelesso et al. [**22**] conducted an 8-months study on 105 patients in a hospital in Bangui and demonstrated that post-stroke depression occurred in 58% of the male patients, and the mean age of the subjects with post-stroke depression was 49.1 years, ranging from 33 to 76 years. Other studies, however, showed a higher incidence of post-stroke depression among women [**23**,**24**]. Variables related to origin showed that 49 (60%) patients were from urban areas and 33 (40%) from rural areas. These differences can be explained by the fact that patients residing in urban areas have greater access to medical services.

The prevalence of post-stroke depression can reach values of up to 50% in the 5 years following vascular events [**25**]. Severe depressive episodes occur with approximately the same frequency as minor depressive episodes. In our study, severe depression was diagnosed in approximately 25% of the subjects.

Patients with post-stroke depression have affected the quality of life [**26**], the same thing being supported by our study (p<0.001).

In our study, acute ischemic lesions located in the left hemisphere, the frontal lobe or the basal ganglia were independent parameters associated with severe depression. Robinson et al. [**27**] and Starkstein et al. [**28**] reported a significantly higher incidence of depression in ganglia on the left side than in patients with other stroke locations. Most recent studies have not demonstrated this association, but Rajashekaran et al. [**29**], Alajbegovic et al. [**30**] and Wei et al. [**31**] described the same associations as we did in our study. These associations were more visible in the first two months after a stroke. Some studies have determined that there is a strong lateralization of emotions in the brain (Davidson RJ, Irwin W.) [**32**].

Higher NIHSS scores were associated with severe depression. Thus, a patient with an NIHSS score over 11 had a 9.4-fold higher probability of experiencing severe depression than a patient with an NIHSS score below 11. The NIHSS assessment can predict the long-term prognosis and the cognitive-behavioral evolution of stroke patients. Alajbegovic et al. and Dąbrowska-Bender et al. demonstrated the value of the NIHSS score for the assessment of the severity of post-stroke depression.

The limitation of the study was the low number of patients. 

## Conclusion

The location of a stroke and the NIHSS score could be related to the severity of the post-stroke depression.

**Conflicts of interest**

The authors declare no conflict of interest.

**Patient consent**

Consent was obtained from each patient.

**Acknowledgments**

The authors would like to thank the proper authorities of the Neurology Clinic of Cluj-Napoca for the assistance. The authors would also like to thank all the participants in this study.
